# Oxypnictide SmFeAs(O,F) superconductor: a candidate for high–field magnet applications

**DOI:** 10.1038/srep02139

**Published:** 2013-07-04

**Authors:** Kazumasa Iida, Jens Hänisch, Chiara Tarantini, Fritz Kurth, Jan Jaroszynski, Shinya Ueda, Michio Naito, Ataru Ichinose, Ichiro Tsukada, Elke Reich, Vadim Grinenko, Ludwig Schultz, Bernhard Holzapfel

**Affiliations:** 1 Institute for Metallic Materials, IFW Dresden, 01171 Dresden, Germany; 2 Applied Superconductivity Center, National High Magnetic Field Laboratory, Florida State University, 2031 East Paul Dirac Drive, Tallahassee, Florida 32310, USA; 3 Department of Applied Physics, Tokyo University of Agriculture and Technology, Koganei, Tokyo 184-8588, Japan; 4 Central Research Institute of Electric Power Industry, 2-6-1 Nagasaka, Yokosuka, Kanagawa 240-0196, Japan

## Abstract

The recently discovered oxypnictide superconductor SmFeAs(O,F) is the most attractive material among the Fe-based superconductors due to its highest transition temperature of 56 K and potential for high-field performance. In order to exploit this new material for superconducting applications, the knowledge and understanding of its electro-magnetic properties are needed. Recent success in fabricating epitaxial SmFeAs(O,F) thin films opens a great opportunity to explore their transport properties. Here we report on a high critical current density of over 10^5^ A/cm^2^ at 45 T and 4.2 K for both main field orientations, feature favourable for high-field magnet applications. Additionally, by investigating the pinning properties, we observed a dimensional crossover between the superconducting coherence length and the FeAs interlayer distance at 30–40 K, indicative of a possible intrinsic Josephson junction in SmFeAs(O,F) at low temperatures that can be employed in electronics applications such as a terahertz radiation source and a superconducting Qubit.

Among the recently discovered Fe-based superconductors^1^, the highest superconducting transition temperature *T*_c_ of 56 K has been reported in SmFeAs(O,F)^2^. This new class of material shows very high upper critical fields at low temperatures together with a moderate anisotropy ranging from 4 to 7^3^, which is suitable for high-field magnet applications. Hence several attempts on wire fabrication using SmFeAs(O,F) by powder-in-tube technique (PIT) have already been reported^4^, despite the lack of information on the field and orientation dependence of intra-grain critical current density [i.e., *J*_c_(*H*, Θ)]. In order to exploit this material class, the knowledge of these properties should be clarified.

Epitaxial thin films are favourable for electronics device applications and investigating transport as well as optical properties thanks to their geometry. Recent success in fabricating epitaxial Fe-based superconducting thin films opens a great opportunity for investigating their physical properties and exploring possible superconducting applications. To date, high-field transport properties of Co-doped SrFe_2_As_2_ (Sr-122) and BaFe_2_As_2_ (Ba-122), and Fe(Se,Te) epitaxial thin films have been reported by several groups^5^,^6^,^7^. For Co-doped Ba-122, *J*_c_ performance can be tuned by introduction of artificial pinning centers and proton irradiation^8^,^9^. Additionally, multilayer approaches that can tailor superconducting properties and their anisotropy have been reported by Lee *et al*^10^. Furthermore, epitaxial Co-doped Ba-122 and Fe(Se,Te) thin films have been realised on ion beam assisted deposition MgO coated conductor templates^11^,^12^,^13^ and the rolling-assisted biaxially textured substrate^14^, respectively. Similarly, high performance K-doped Ba-122 and Sr-122 wires by PIT have been reported by Weiss *et al.*^15^ and Gao *et al*^16^, respectively. These results are very promising for realising Fe-based superconducting high-field applications. However, transport critical current properties of high-*T*_c_ (i.e., over 50 K) oxypnictide thin films have not been reported before due to the absence of high quality films. Recently, *in situ* prepared *Ln*FeAs(O,F) (*Ln* = Nd and Sm) epitaxial thin films with *T*_c_ exceeding 50 K have been realised by molecular beam epitaxy (MBE)^17^,^18^. These successes give many possibilities to explore electro-magnetic properties.

In this paper, we report on various *in – plane* (i.e., current is flowing on the crystallographical *ab*-plane) transport properties up to 45 T of epitaxial SmFeAs(O,F) thin films grown by MBE on CaF_2_ (001) single crystalline substrates and discuss their pinning properties. A high *J*_c_ of over 10^5^ A/cm^2^ was recorded at 45 T and 4.2 K for both crystallographic directions, which is favourable for high-field magnet applications. By analysing pinning properties the dimensional crossover between the out-of-plane superconducting coherence length *ξ*_c_ and the Fe-As interlayer distance *d*_FeAs_ was observed at 30–40 K. This indicates the possible intrinsic Josephson junction in SmFeAs(O,F) at low temperatures.

## Results

### Microstructural analyses

**Figure 1:**
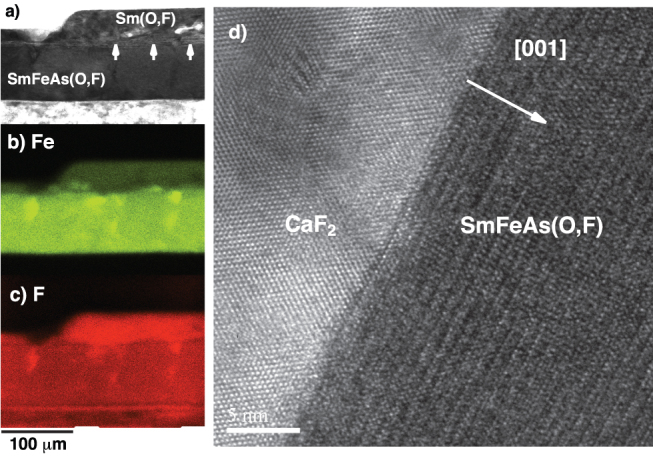
Microstructural analyses by TEM. (a) Cross-sectional scanning TEM image of the SmFeAs(O,F) thin film. A crystallographically disordered layer as indicated by the arrows is present between Sm(O,F) cap and SmFeAs(O,F) layers. (b) Elemental Fe and (c) F mappings measured by energy dispersive x-ray spectroscopy. (d) High-resolution TEM image of the SmFeAs(O,F) thin film in the vicinity of the CaF_2_ substrate/SmFeAs(O,F) film interface.

As verified by x-ray diffraction, the biaxially textured SmFeAs(O,F) film with a narrow full width at half maximum (FWHM) of less than 0.65° was obtained (See in Supplementary Fig. S1). As shown in Fig. 1a, trapezoid shaped Sm(O,F) cap layers, which are changed from SmF_3_, are aligned discontinuously. Additionally, a crystallographically disordered layer with around 20 nm thickness as indicated by the arrows is present between Sm(O,F) cap and SmFeAs(O,F) layers. Relatively dark particles are observed in the SmFeAs(O,F) matrix, which are identified as iron-fluoride, presumably FeF_2_, by elemental mappings shown in Figs. 1b and 1c. This is due to the excess of Fe supplied during the film growth.

Compared to Fe(Se,Te)^19^ and Co-doped Ba-122 films^20^ grown by pulsed laser deposition, a relatively sharp and clean interface is observed between SmFeAs(O,F) and CaF_2_ substrate, as shown in Fig. 1d. Furthermore, SmFeAs(O,F) layers contained neither correlated defects nor large angle grain boundaries (GBs).

### Resistivity measurements up to 45 T

**Figure 2:**
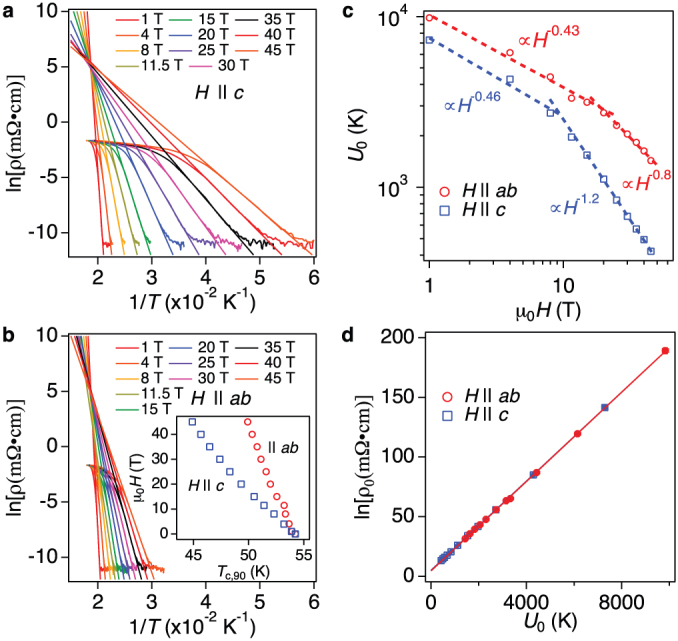
In-field resistivity (*ρ*) measurements of SmFeAs(O,F) film up to 45 T and the analyses of the activation energy of pinning potential (*U*_0_). (a) Arrhenius plots of *ρ* at various magnetic fields parallel to the crystallographic *c*-axis and (b) *ab*-plane. The inset shows the *μ*_0_*H* − *T*_c,90_ diagram of SmFeAs(O,F) film for both directions, which is the identical to the extracted temperature dependence of the upper critical fields by employing a 90% criterion of the normal state resistivity. (c) Field dependence of the activation energy for *H* || *c* and || *ab*. (d) Relationship between ln*ρ*_0_ and *U*_0_ for *H* || *c* and || *ab*.

The superconducting transition temperature *T*_c,90_ defined as 90% of the normal state resistivity is 54.2 K in zero magnetic field. Figures 2a and 2b show the Arrhenius plots of resistivity for both crystallographic directions measured in static fields up to 45 T. For both directions the *T*_c,90_ is shifted to lower temperature with increasing *H*, as shown in the inset of Fig. 2b. The respective *T*_c,90_ at 45 T for *H* || *c* and || *ab* are 44.9 K and 49.9 K. Significant broadening of the transition is observed for *H* || *c*, which is reminiscent of high-*T*_c_ cuprates. Such broadening of the transition originates from enhanced thermally activated vortex motion for *H* || *c*. In contrast, the in-field *T*_c,90_ as well as its transition width for *H* || *ab* are less affected by *H* than that for *H* || *c*.

The activation energy *U*_0_(*H*) for vortex motion can be estimated by the model of thermally activated flux flow^21^. On the assumption that *U*(*T*, *H*) = *U*_0_(*H*)(1 − *T*/*T*_c_), we obtain ln*ρ*(*T*, *H*) = ln*ρ*_0_(*H*) − *U*_0_(*H*)/*T* and ln*ρ*_0_(*H*) = ln*ρ*_0f_ + *U*_0_(*H*)/*T*_c_, where *ρ*_0f_ is the prefactor. In Figs. 2a and 2b, the slope of linear fits corresponds to the *U*_0_ for vortex motion. Figure 2c shows *U*_0_ as a function of *H* for both major directions. It can be seen that *U*_0_(*H*) shows a power law [i.e., *U*_0_(*H*) ~ *H*^−*α*^] for both crystallographic directions. In the range of 1 < *μ*_0_*H* < 8 T, *α* = 0.46 is observed for *H* || *c*, whilst a similar field dependence of *U*_0_(*H*) reaches 20 T for *H* || *ab*. In higher fields *U*_0_ for *H* || *ab* shows a weak *H* dependence. On the other hand, *α* = 1.2 is obtained for *H* || *c* in the range of 8 < *μ*_0_*H* < 45 T, which is close to 1, suggesting a crossover from plastic to collective pinning at around *μ*_0_*H* ~ 8 T^22^.

Figure 2d shows the relationship between ln*ρ*_0_ and *U*_0_ for *H* || *c* and || *ab*. The linear fitting for *H* || *c* yields *T*_c_ = 53.4 ± 0.2 K, whilst the corresponding value for *H* || *ab* is *T*_c_ = 53.5 ± 0.2 K. Both *T*_c_ values are equal within error and close to *T*_c,90_. This perfect linear scaling is due to the correct assumption that both *U*(*T*, *H*) = *U*_0_(*H*)(1 − *T*/*T*_c_) and *ρ*_0f_ = const. conditions are satisfied in a wide temperature range in Figs. 2a and 2b.

### In-field *J*_c_ performance

**Figure 3:**
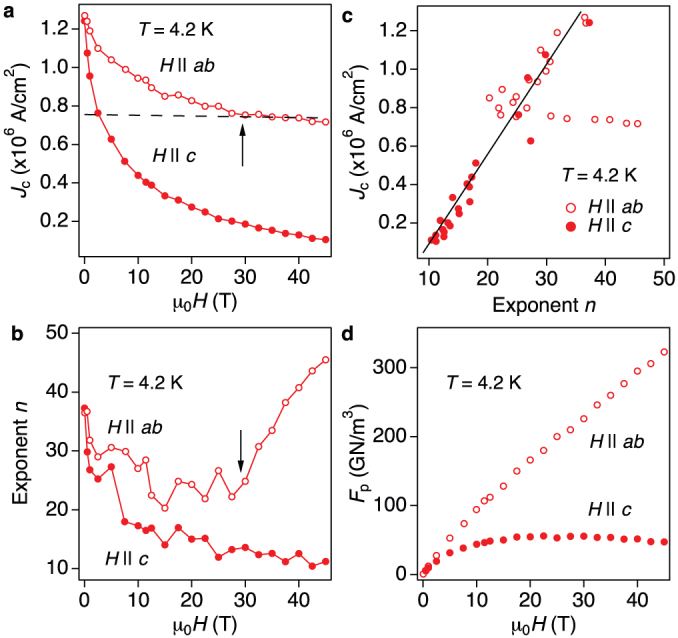
In-field critical current density (*J*_c_) performance of SmFeAs(O,F) thin film at 4.2 K. (a) Field dependence of *J*_c_ measured at 4.2 K up to 45 T for both crystallographic directions and (b) the corresponding exponent *n* values.A crossover from extrinsic to intrinsic pinning is shown by the arrow. (c) Scaling behaviour of the field dependent *J*_c_. (d) The pinning force density *F*_p_ for both crystallographic directions at 4.2 K.

The field dependence of *J*_c_ at 4.2 K for both principal crystallographic directions measured up to 45 T is displayed in Fig. 3a. *J*_c_ for 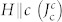 is lower than that for 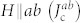, which is a consequence of moderate anisotropy of SmFeAs(O,F). This tendency is observed for all temperature regions (see Supplementary Fig. S2). It is worth mentioning that a 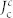 of over 10^5^ A/cm^2^ was recorded even at 45 T, which is favourable for high-field magnet applications.


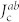
 is observed to decrease gradually with *H* and it shows an almost constant value of 7.4 × 10^5^ A/cm^2^ for μ_0_*H* > 28 T. This behaviour can be explained by a combination of extrinsic (i.e., normal precipitates and stacking faults) and intrinsic pinning, which is a similar observation in quasi two-dimensional (2D) system YBa_2_Cu_3_O_7−δ_ [i.e., 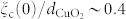, where *ξ*_c_(0) is the out-of-plane superconducting coherence length at zero temperature and 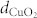 is the interlayer distance between CuO_2_ planes]^23^. SmFeAs(O,F) is an alternating structure of SmO and FeAs layers, similarly to high-*T*_c_ cuprates. Additionally, *ξ*_c_(0) is shorter than the interlayer distance between Fe-As planes *d*_FeAs_. Hence, modulation of superconducting order parameter along the crystallographic *c*-axis (i.e., intrinsic pinning) is highly expected in SmFeAs(O,F). In fact the extrinsic pinning is dominant up to 28 T, whereas the intrinsic pinning overcomes the extrinsic one above 28 T. The estimation of *ξ*_c_(0) and *d*_FeAs_ in our SmFeAs(O,F) case will be discussed later.

By analysing the *E-J* curves from which *J*_c_ was determined, we obtain the information on the pinning potential. On the assumption of a logarithmic current dependence of the pinning potential *U*_p_ for homogeneous samples, *E-J* curves show a power-law relation *E~J^n^* (*n* ~ *U*_p_/*k*_B_*T*, where *k*_B_ is the Boltzmann constant)^24^. Hence *J*_c_ scales with *n* and indeed the field dependence of *n* has a similar behaviour to *J*_c_(*H*) for *H* || *c*, as presented in Fig. 3b. For *H* || *ab*, *n* decreases with *H* up to 28 T, similarly to the *J*_c_(*H*) behaviour, whereas at larger field it suddenly increases due to the dominating intrinsic pinning. Hence a failure to scale *J*_c_ with *n* or deviations as shown in Fig. 3c indicates the presence of intrinsic pinning.

The field dependence of the pinning force density *F*_p_ for both crystallographic directions at 4.2 K is summarised in Fig. 3d. An almost field independent *F*_p_ above 10 T for *H* || *c* is observed, whereas *F*_p_ for *H* || *ab* is still increasing up to the maximum field available.

### Angular dependence of *J*_c_

**Figure 4:**
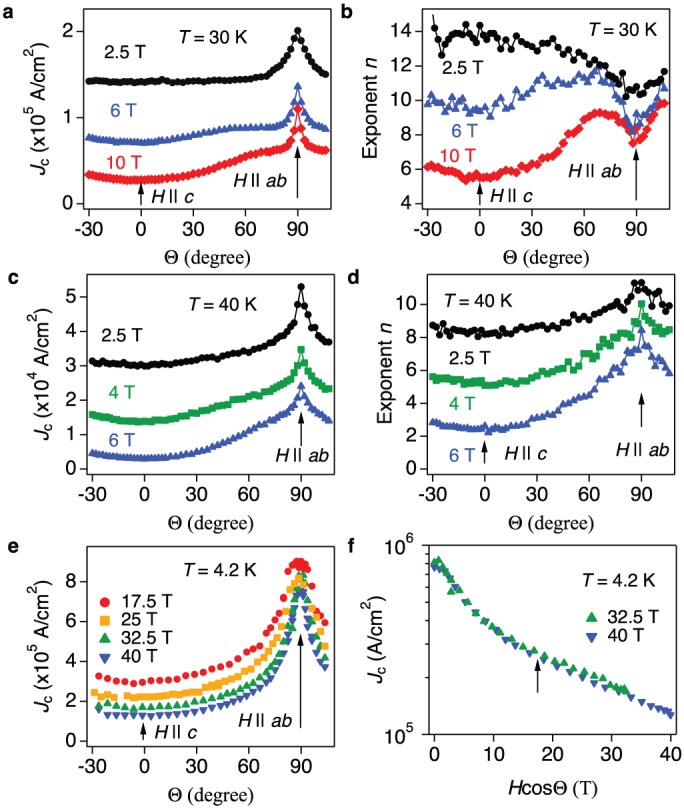
Field and orientation dependence of critical current density (*J*_c_) of SmFeAs(O,F) thin film. (a) Angular dependence of *J*_c_ measured at 3 different applied magnetic fields at 30 K and (b) the corresponding exponent *n* values. (c) *J*_c_(Θ, *H*) measured at 40 K under several magnetic fields (*μ*_0_*H* = 2.5, 4 and 6 T) and (d) the corresponding exponent *n* values. (e) Angular dependence of *J*_c_ at 4.2 K under various applied magnetic fields up to 40 T. (f) Scaling behaviour of the angular dependent *J*_c_ measurements. Below 17 T (i.e., by substituting *μ*_0_*H* = 32.5 T and Θ = 59° in *H* cosΘ) as indicated by the arrow, both curves overlap each other.

In order to gain a deeper insight into the flux pinning, the angular dependence of *J*_c_ [*J*_c_(Θ), where Θ is the angle between *H* and the *c*-axis] was measured and summarised in Fig. 4. Figure 4a presents *J*_c_(Θ) at 30 K in three different magnetic fields. Almost isotropic *J*_c_(Θ, 2.5 T) of around 0.14 MA/cm^2^ was observed at angles Θ up to 75°. Similar isotropic behaviour is seen at 6 T. These results suggest the presence of *c*-axis correlated defects. However, the presence of these defects is ruled out by TEM investigation, since only relatively large FeF_2_ particles are observed in the SmFeAs(O,F) matrix. Recently, van der Beek *et al.* pointed out that defects of size larger than the out-of-plane coherence length contribute to *c*-axis pinning in anisotropic superconductors^25^. Additionally, the intrinsic pinning is active below *T* = 30 ~ 40 K, as shown below. Hence the combination of large particles and the intrinsic pinning may be responsible for this isotropic *J*_c_(Θ).

For *H* || *ab*, a broad maximum of *J*_c_ is observed and this peak becomes sharper with increasing *H* (Fig. 4a). However, the corresponding *n* shows a broad minimum for *H* close to *ab* direction (Fig. 4b), which is opposite behaviour to *J*_c_. This is due to the thermal fluctuation of Josephson vortices, which leads to flux creep. Here, the flux creep rate *S* = −*dln*(*J*)/*dln*(*t*) and the exponent *n* are related as *S* = 1/(*n* − 1)^26^. When the applied field is close to the *ab*-plane, a number of thermally fluctuated Josephson vortices are generated, leading to an increase in *S.* This could quantitatively explain a dip of *n* at around *H* close to *ab*. Similar behaviour has been observed in YBa_2_Cu_3_O_7−*δ*_ thin films^27^,^28^,^29^ and Fe(Se,Te) thin films^30^. On the other hand, this dip of *n* disappears at 40 K, although the *J*_c_ still shows a broad maximum (Figs. 4c and 4d). Hence the activation temperature of the intrinsic pinning is between 30 and 40 K, which is in good agreement with the transition temperature between Abrikosov-and Josephson-like vortices in SmFeAs(O,F) single crystals^31^.

Figure 4e shows *J*_c_(Θ) measured at 4.2 K in fields up to 40 T. A sharp peak is observed for *H* || *ab* with a *J*_c_ of around of 8 × 10^5^ A/cm^2^. For 2D superconductors (e.g., Bi_2_Sr_2_CaCu_2_O_8+*x*_), the relation 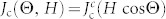 holds in the intrinsic pinning regime, whereas 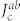 is field independent^32^,^33^. Thus, in this regime *J*_c_(Θ, *H*) depends only on the field component along the *c*-axis. For our SmFeAs(O,F) thin film, the aforementioned condition is satisfied above 28 T at which the crossover field between extrinsic and intrinsic pinning is observed (see Fig. 3a). Hence, for Θ > 59° (sinΘ = 28/32.5 = 0.86, Θ = sin^−1^(0.86) = 59°) the *ab* component of the applied fields exceed 28 T, entering in the field-independent 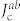 region. It means that both angular–*J*_c_ curves measured at 32.5 and 40 T rescale with *H* cosΘ, as shown in Fig. 4f.

## Discussion

We estimate the *ξ*_c_(0) by using *T*_cr_ = (1 − *τ*_cr_)*T*_c_, where *T*_cr_ is the dimensional crossover temperature and 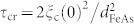 is the dimensionless ratio characterising the crossover from quasi-2D layered to continuous 3D anisotropic behaviour^34^. By substituting *T*_cr_ = 30–40 K and *d*_FeAs_ = 0.858 nm from the x-ray diffraction shown in Supplementary Fig. S1, 
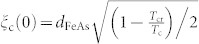
 is calculated to 0.3~0.4 nm. The ratio *ξ*_c_(0)/*d*_FeAs_ = 0.35 ~ 0.47 explains the intrinsic pinning related to a quasi 2D system observed in this film. The relation 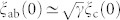 yields *ξ*_ab_(0) = 1.7 ~ 2.2 nm, where *γ* is the effective-mass or resistivity anisotropy, which is about 30 at *T* = 0 K from measurements of the *c*-axis plasma frequency using infrared ellipsometry^35^. The evaluated superconducting coherence lengths for both crystallographic directions are in very good agreement with single-crystals values reported by Welp *et al*^36^.

The presence of a dimensional crossover indicates a possible intrinsic Josephson junction in SmFeAs(O,F), which can be used in superconducting electronics applications such as a terahertz radiation source and a superconducting Qubit^37^,^38^. Indeed, the intrinsic Josephson junction was reported for a PrFeAsO_0.7_ single crystal, where an *s*-shaped stack junction in *c*-direction was prepared by focused ion beam^39^.

For high-field magnet applications, a high *J*_c_ together with a low *J*_c_ anisotropy (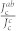) in the presence of magnetic field is necessary. The present results are promising, since *J*_c_ is over 10^5^ A/cm^2^ at 45 T for both crystallographic directions. Further increasing in *J*_c_ is possible, since the only appreciable defects in our SmFeAs(O,F) films are large FeF_2_ particles. Improved pinning performance and, as a consequence, larger *J*_c_ could be realised by incorporating artificial pinning centres similarly to Co-doped Ba-122 thin films reported by Tarantini *et al*^8^. Albeit the *J*_c_ anisotropy is increasing with *H*, this value is still low compared to high-*T*_c_ cuprtaes. For instance, *J*_c_ anisotropy is about 3.6 at 30 T and 4.2 K in SmFeAs(O,F), whereas the corresponding value in YBa_2_Cu_3_O_7−*δ*_ is over 7, albeit the latter shows higher *J*_c_ than the former^40^.

PIT is a more realistic process than MBE for high-field magnet applications. High temperature heat treatment in PIT leads to a loss of F, however, this problem can be solved by employing a low temperature synthesis and ex-situ process with SmF_3_ containing binder as explained in refs. 41,42. Despite a high *T*_c_ of over 45 K for both SmFeAs(O,F) wires, self-field *J*_c_ shows only a few thousand A/cm^2^ at 4.2 K, which is presumably due to grain boundaries (GBs), poor grain connectivity and low density. Obviously these PIT processed wires contain a high density of large angle GBs. In the case of Co-doped Ba-122 GBs with misorientation angles above 9° seriously reduce the critical current^43^. However, PIT processed K-doped Ba-122 and Sr-122 wires showed a relatively high inter-grain *J*_c_^15^,^16^. Clean GBs (i.e., no segregation of secondary phases around GBs), good grain connectivity and a low anisotropy may be responsible for these high performance wires. An approach similar to the one employed in K-doped Ba-122 and Sr-122 wires fabrication may be useful for improving inter-grain *J*_c_ in SmFeAs(O,F) wires as well. Nevertheless bicrystal experiments on SmFeAs(O,F) will give a valuable information on these issues.

To conclude, we have explored intrinsic electro-magnetic properties of epitaxial SmFeAs(O,F) thin films prepared by MBE on CaF_2_ (001) substrate by measuring field-angular dependence of transport properties up to 45 T. Our findings strongly support the presence of a competition behaviour between extrinsic pinning below 28 T and intrinsic pinning above 28 T. We also determined that the intrinsic pinning starts being effective below *T* = 30 ~ 40 K, at which the crossover between the out-of-plane coherence length and the interlayer distance occurs. This knowledge of SmFeAs(O,F) electro-magnetic properties could stimulate future development of superconducting applications of this class of material.

## Methods

### Epitaxial SmFeAs(O,F) film preparation by MBE

SmFeAs(O,F) films of 80 nm thickness have been grown in the customer-designed MBE chamber. A parent compound of SmFeAsO film was prepared on CaF_2_ (001) single crystalline substrate at 650°C, followed by the deposition of a SmF_3_ cap layer. Empirically, Fe-rich pnictide films fabricated by MBE showed high *J*_c_ values^44^. Hence a slight Fe excess was supplied during the growth of SmFeAsO layers. After the overlayer deposition, the sample was kept at the same temperature in the MBE chamber for 0.5 h for the purpose of F diffusion into the SmFeAsO layer. The detailed fabrication process can be found in ref. 17. SmFeAs(O,F) films are grown epitaxially with high crystalline quality confirmed by x-ray diffraction, which is summarised in Supplementary Fig. S1.

### Microstructural analyses by TEM

A TEM lamella was prepared by means of focused ion beam. Microstructural analyses have been performed by using a JEOL TEM-2100F transmission electron microscope equipped with an energy-dispersive x-ray spectrometer.

### In-plane transport properties measurement

A small bridge of 70 *μ*m width and 0.7 mm length was fabricated by laser cutting. *I-V* characteristics on this sample were measured with four-probe configuration by a commercial physical property measurement system [(PPMS) Quantum Design] up to 12 T. Transport measurements up to 45 T were carried out in the high field dc facility at the National High Magnetic Field Laboratory (NHMFL) in Tallahassee, FL. A voltage criterion of 1 *μ*V/cm was employed for evaluating *J*_c_. The magnetic field *H* was applied in maximum Lorentz force configuration during all measurements (*H* ⊥ *J*, where *J* is current density).

## Author Contributions

K.I., J.H. and C.T. designed the study and wrote the manuscript together with M.N., J.J., I.T., V.G., L.S. and B.H. Thin films were prepared by S.U., K.I. and S.U. conducted x-ray experiments. C.T. and K.I. measured low field transport properties. C.T., K.I., J.H., F.K., M.N. and J.J. investigated high-field transport properties. A.I., I.T. and E.R. conducted TEM investigation. All authors discussed the results and implications and commented on the manuscript.

## Supplementary Material

Supplementary Information Supplementary Information for "Oxypnictide SmFeAs(O,F) superconductor: a candidate for high-field magnet applications"
