# Supplementary material for: Oxypnictide SmFeAs(O,F) superconductor: a candidate for high–field magnet applications
Source: Sci Rep. 2013 Jul 4;3:2139. doi: 10.1038/srep02139 (PMC3705587; doi:10.1038/srep02139)
Supplement: Supplementary Information — for "Oxypnictide SmFeAs(O,F) superconductor: a candidate for high-field magnet applications" [file srep02139-s1.pdf]

Supplementary Information for "Oxypnictide SmFeAs(O,F) superconductor: a candidate for high–field magnet applications"

KAZUMASA IIDA[1,*], JENS HÄNISCH[1], CHIARA TARANTINI[2], FRITZ KURTH[1], JAN JAROSZYNSKI[2], SHINYA UEDA[3], MICHIO NAITO[3], ATARU ICHINOSE[4], ICHIRO TSUKADA[4], ELKE REICH[1], VADIM GRINENKO[1], LUDWIG SCHULTZ[1], AND BERNHARD HOLZAPFEL[1]

1. Institute for Metallic Materials, IFW Dresden, 01171 Dresden, Germany

2. Applied Superconductivity Center, National High Magnetic Field Laboratory, Florida State University, 2031 East Paul Dirac Drive, Tallahassee, Florida 32310, USA

3. Department of Applied Physics, Tokyo University of Agriculture and Technology, Koganei, Tokyo 184-8588, Japan

4. Central Research Institute of Electric Power Industry, 2-6-1 Nagasaka, Yokosuka, Kanagawa 240-0196, Japan

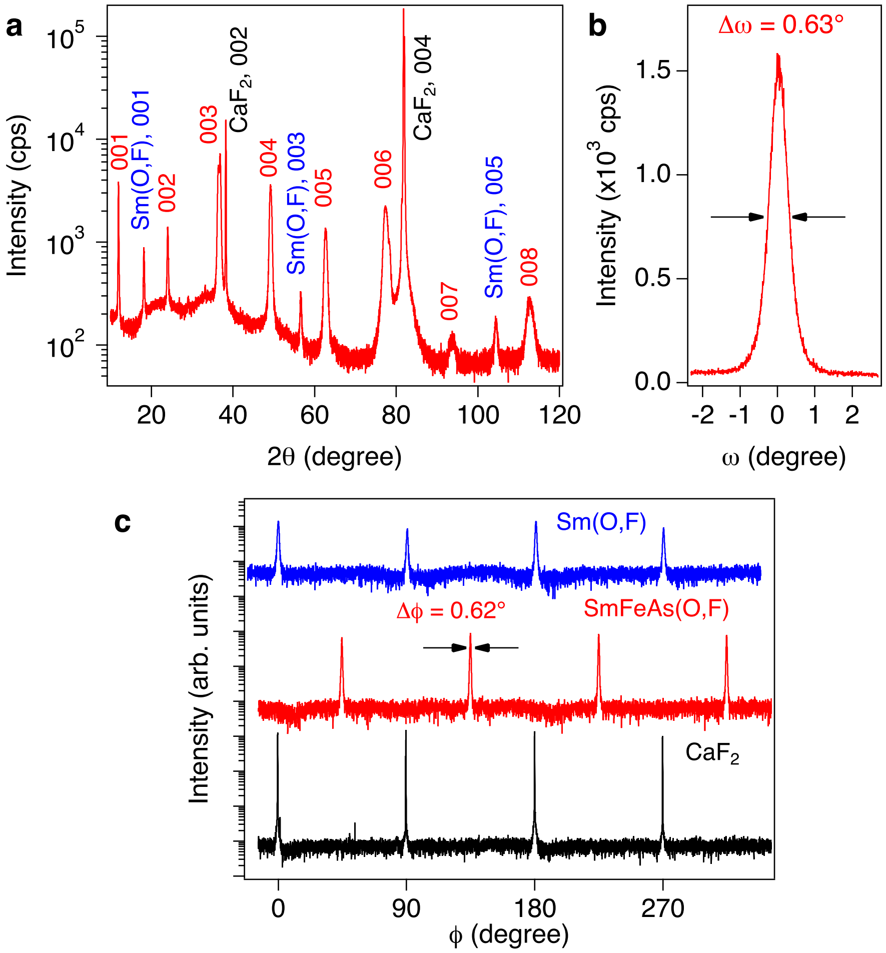

FIGURE S1. (a) The $\theta/2\theta$ scan of the SmFeAs(O,F) thin film grown on CaF_2 (001) substrate. All peaks are assigned as the $00l$ reflections of SmFeAs(O,F), Sm(O,F) and CaF_2, indicative of the c-axis textured for both SmFeAs(O,F) and Sm(O,F) layers. (b) The rocking curve of the 004 reflection of SmFeAs(O,F) shows a narrow full width at half maximum (FWHM) of 0.63°, proving a highly out-of-plane textured film. (c) ϕ scans of the 112 peak of SmFeAs(O,F), the 112 peak of Sm(O,F), and the 111 peak of CaF_2. The ϕ scan of SmFeAs(O,F) revealed no additional reflections other than sharp (average FWHM, $\Delta\phi$, of 0.63°) and strong reflections at every 90°, indicative of biaxial texture. These results highlight that the SmFeAs(O,F) is grown epitaxially with high crystalline quality. Interestingly Sm(O,F) cap layer is also grown biaxial textured. The epitaxial relation of each layer and substrate is confirmed to (001)[100]Sm(O,F)$\|$(001)[110]SmFeAs(O,F)$\|$(001)[100]CaF_2.

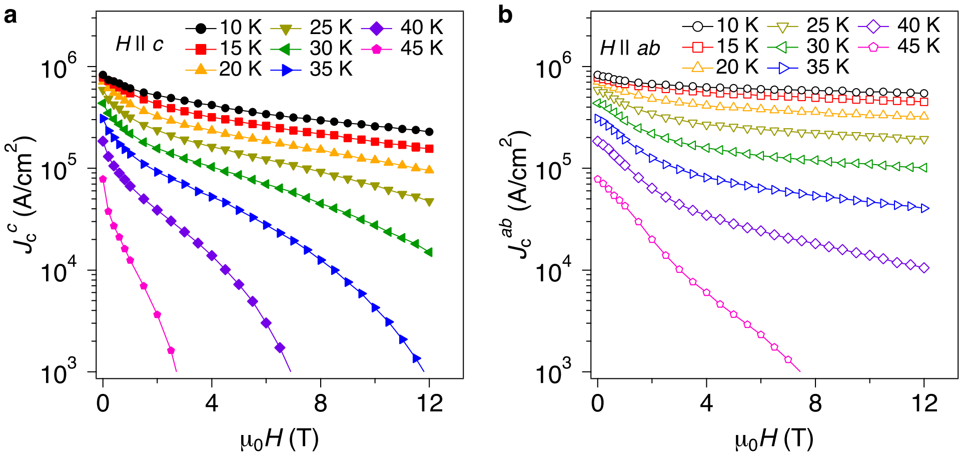

FIGURE S2. Magnetic field dependence of J_c measured at different temperatures for (a) $H \parallel c$ (J_c^c) and (b) $H \parallel ab$ (J_c^{ab}). For $\mu_0 H > 3\,\mathrm{T}$, J_c^{ab} is getting more and more insensitive to H with decreasing temperature.
